# Factors associated with long-term gastrointestinal symptoms in colorectal cancer survivors in the women’s health initiatives (WHI study)

**DOI:** 10.1371/journal.pone.0286058

**Published:** 2023-05-19

**Authors:** Claire J. Han, Kerryn W. Reding, Matthew F. Kalady, Rachel Yung, Heather Greenlee, Electra D. Paskett

**Affiliations:** 1 Center for Healthy Aging, Self-Management and Complex Care, Ohio State University, College of Nursing, Columbus, OH, United States of America; 2 Department of Cancer Control Survivorship, Ohio State University Comprehensive Cancer Center, Columbus, OH, United States of America; 3 Department of Biobehavioral Nursing and Health Informatics, University of Washington, School of Nursing, Seattle, WA, United States of America; 4 Public Health Sciences Division, Fred Hutchinson Cancer Research Center, Seattle, WA, United States of America; 5 Division of Colon and Rectal Surgery, Clinical Cancer Genetics Program, Ohio State University Comprehensive Cancer Center, Columbus, OH, United States of America; 6 Fred Hutchinson Cancer Research Center, Clinical Research Division, University of Washington School of Medicine, Medical Oncology, Seattle, WA, United States of America; 7 Cancer Prevention Program, Public Health Sciences and Clinical Research Divisions, Fred Hutchinson Cancer Center, Seattle, WA, United States of America; 8 Department of Medical Oncology, University of Washington School of Medicine, Seattle, WA, United States of America; 9 Department of Internal Medicine in the College of Medicine, Ohio State University, Columbus, OH, United States of America; 10 Division of Epidemiology in the College of Public Health, Ohio State University, Columbus, OH, United States of America; Brazilian national cancer institute, BRAZIL

## Abstract

**Purpose:**

Colorectal cancer (CRC) survivors often experience long-term symptoms after cancer treatments. But gastrointestinal (GI) symptom experiences are under-investigated in CRC survivors. We described persistent GI symptoms after cancer treatments in female CRC survivors and assessed GI symptoms’ risk and life-impact factors.

**Methods:**

A cross-sectional study utilized data from the Women’s Health Initiative (WHI) Life and Longevity After Cancer (LILAC) study that recruited postmenopausal women. Correlation analyses and multivariable linear regression models were used.

**Results:**

CRC survivors after cancer treatments were included (N = 413, mean age 71.2 years old, mean time since diagnosis = 8.1 years). 81% of CRC survivors experienced persistent GI symptoms. Bloating/gas was the most prevalent (54.2%± 0.88) and severe GI symptom, followed by constipation (44.1%±1.06), diarrhea (33.4%±0.76), and abdominal/pelvic pain (28.6%±0.62). Significant risk factors for GI symptoms include time since cancer diagnosis (<5 years), advanced cancer stage, high psychological distress, poor dietary habits, and low physical activity. Fatigue and sleep disturbance were the most significant risk factors for long-term GI symptoms (β = 0.21, t = 3.557; β = 0.20, t = 3.336, respectively, *Ps* < .001). High severity of GI symptoms was positively associated with poor quality of life (QOL), increased daily life interferences (social and physical functions), and low body image satisfaction (*Ps* < .001).

**Conclusions:**

Women CRC survivors experience a high GI symptom burden, highlighting the need to inform policy and improve the QOL of cancer survivors. Our findings will aid in identifying those more vulnerable to symptoms, and inform future survivorship care interventions (i.e., community-based cancer symptom management) by considering multiple risk factors (e.g., psychological distress).

## Introduction

Colorectal cancer (CRC) is the third most commonly diagnosed cancer in men and women worldwide and the second leading cause of cancer death in the United States (U.S.) [1]. With the increased survival rate of CRC survivors (the 5-year survival rate in CRC = 64% for combined cancer stages in 2020 in the U.S.), many are living with the long-term impacts of cancer treatments. CRC survivors face the greatest challenge in dealing with residual symptoms, which influence daily routines of life, function, financial burden, health-related quality of life (HRQOL), and work limitations [2, 3].

In particular, CRC survivors often reported gastrointestinal (GI) symptoms [3–5]. Common cancer-related GI symptoms include bloating, nausea, vomiting, diarrhea, constipation, and abdominal pain in CRC survivors [6–8]. Given that most CRC survivors underwent surgery, chemotherapy, radiation, or combined, the GI symptoms in CRC survivors may arise either from the disease in the GI tract or as a side effect following cancer treatments [6, 7]. Long-term intestinal injury with endothelial damage, inflammation fibrosis, ischemia, and necrosis in CRC survivors may present clinically as severe damage to the GI tract as strictures and stenosis with obstruction, fistulas, and bowel perforation, ultimately linked to systematic health problems (i.e., sepsis, malnutrition) [6, 9]. The health and economic burden of GI symptoms after cancer treatments are significant. Treatment-related GI side effects often result in increased medical room visits, unplanned medical consultation, hospitalization, or cancer treatment dose reduction or discontinuation of therapy, limiting the optimal efficacy of cancer treatments [4, 9, 10].

While cancer treatments contribute to increasing cancer survival rates in CRC survivors, little research has been devoted to understanding the impact of cancer treatments on persistent GI symptoms in long-term female CRC survivors. For both men and female CRC survivors, the importance of GI symptoms in CRC survivors has received less attention than other cancer-related symptoms, such as psychological distress, peripheral neuropathy, or cognitive impairment [4]. Most studies that have examined symptoms have focused on psychological distress or breast cancer survivors [2, 3, 8]. In terms of research examining GI symptoms in CRC survivors, evidence is mainly limited by acute GI symptoms during or right after cancer treatments [4, 11, 12], or there is a lack of understanding of factors relating to long-term GI symptoms in CRC survivors [13–15].

Notably, sex differences may exist in symptom experiences in CRC survivors. The influence of sex has been reported in symptom experiences, as women tend to report more symptoms than men in the general population [16]. Sex differences also affect the severity, as women have a 1.5 times higher risk of severe side effects of cancer treatments than male CRC survivors [17]. Research points to the role of genetic differences and female sex hormones in the greater report of symptoms in female CRC survivors [17]. However, few studies have been performed to understand long-term GI symptoms in female CRC survivors. In previous research examining symptoms in CRC survivors [4, 13, 14], most participants were men (65% across the studies). There is limited data investigating why some patients with CRC suffer from severe long-term GI symptoms and others do not. Relatedly, another area requiring attention is to elucidate which patients will recover from GI symptoms while others will carry on having long-term GI symptoms. A significant gap in the field is the understanding of the long-term symptom impacts of cancer treatment in female CRC survivors—primarily due to the challenges of systematically collecting treatment data in patients followed for long-term outcomes (as opposed to acute symptoms occurring at the time of treatment).

One exception is the Women’s Health Initiative (WHI), a study of 160,000+ postmenopausal women, which launched the Life and Longevity After Cancer (LILAC) study to follow the 12,000+ WHI participants who developed cancer since enrollment. The WHI LILAC study data provide a unique opportunity to efficiently address research gaps with a particular focus on clinical impact, which allows for identifying CRC survivors who are most vulnerable to GI symptoms in the long term.

In this paper, we aimed to 1) examine the prevalence and severity of long-term GI symptoms in female CRC survivors, 2) identify potential risk factors, and 3) explore the impact of long-term GI symptoms in female CRC survivors. To our knowledge, this is the first study to characterize long-term GI symptoms and its correlates in community-dwelling long-term female CRC survivors.

## Materials and methods

**Study design, setting, and sample** This is a cross-sectional design study with a secondary data analysis using existing data from the WHI LILAC studies [18, 19]. Full details of WHI LILAC have been published elsewhere [18, 19]. The WHI is a longitudinal, prospective cohort study, which recruited postmenopausal women aged between 50–79 years from 40 clinical sites in the US began in 1993 in either the observational study (OS, n = 93,676, ending in 1993–2005) or the clinical trials (CT, n = 68,132, 1993 to 1998). Participants were subsequently invited to continue in WHI extension studies (ES) in 2005–2010 (ES1) and again in 2010 (ES2). The LILAC was developed to study cancer survivorship as a sub-cohort study within the WHI. Starting in 2013, the LILAC study started to enroll female cancer patients diagnosed with certain types of cancer (colorectal, breast, ovarian/fallopian tube/primary peritoneal, endometrial, lung, melanoma, non-Hodgkin lymphoma, and leukemia) during the WHI [18, 19]. Women with no prior cancer diagnosis at WHI enrollment were eligible for LILAC if they had a confirmed cancer diagnosis during WHI follow-up in the CT or OS.

For this study, we included participants with CRC in the WHI LILAC study who were in the OS and actively participating in the second extension study (ES2). As of September 15, 2015, 796 CRC survivors were enrolled in the LILAC study [18]. Eligibility criteria for this study are the following: 1) CRC diagnosis before 2011, 2) completion of the LILAC Baseline Questionnaire, the LILAC Annual Survey, and the Lifestyle Questionnaire. These WHI LILAC study instruments were validated in 7,760 participants of the WHI study [18]. Because a cancer diagnosis could have occurred at any time before the collection of variables used for this study, the time frame of the cancer diagnosis varied. Only participants in the OS were eligible because of the availability of the necessary questionnaires. For the parent study of this secondary data analysis (i.e., the WHI LILAC study), the WHI project was reviewed and approved by the Fred Hutchinson Cancer Research Center (Fred Hutch) Institutional Review Board (IRB) in accordance with the U.S. Department of Health and Human Services regulations at 45 CFR 46 (approval number: IR# 3467-EXT). Participants provided written informed consent to participate. Additional consent to review medical records was obtained through signed written consent, and the institutional review board approved both studies at each participating institution [18, 19]. The Fred Hutch IRB waived the consent for the current descriptive secondary data analysis.

### Study variables and data resources

#### Baseline measures

*Demographic* and *clinical characteristics data* was collected from the baseline WHI LILAC questionnaire. Comorbidities were assessed using a modified Charlson Comorbidity Index (CCI) score as characterized previously in the WHI [20]. Scores were calculated based on the presence of specific comorbid conditions, such as cardiovascular diseases, chronic obstructive pulmonary disease, and diabetes, using WHI baseline data and self-reported medical history [20]. In the CCI, the scores range from 0 to 15, with higher scores indicating greater morbidity. Based on the CCI score, the severity of each comorbidity was categorized into three grades: mild, with CCI scores of 1–2; moderate, with CCI scores of 3–4; and severe, with CCI scores ≥ 5.

#### GI symptoms

In this study, we included six GI symptoms as the most common GI symptoms in CRC survivors [4]: abdominal/pelvic pain, bloating or gas, constipation, diarrhea, uncontrolled bowel movements, and heartburn. GI symptom questions from the LILAC Annual Survey were scored using a 4-point Likert scale as 0 (*did not occur*), 1 (*mild)*, 2 (*moderate*), and 3 (*severe*) in the past four weeks. We computed a composite GI symptom score (as a primary outcome), calculated by taking the average of six GI symptom items. The GI composite score ranged from 0–3, with 3 indicating a more severe GI symptom. Cancer patients often experience distinct symptoms (e.g., a subgroup with high diarrhea/low constipation, a subgroup with high abdominal pain severity/low diarrhea); and each GI symptom may have its pathophysiological mechanisms requiring different management strategies [21]. Thus, we also analyzed six individual GI symptoms in our analyses. Cronbach’s alpha using our data set was α = .84.

#### Potential factors relating to GI symptoms

*Non-GI symptoms* (i.e., depression, anxiety, fatigue, and sleep), dietary habits, and physical activity were collected from the WHI LILAC Annual Survey. Non-GI symptoms were scored using a 4-point Likert scale from 0 *(did not occur)*, 1 *(mild)*, 2 *(moderate)*, to 3 *(severe)* in the past four weeks. Cronbach’s alpha using our data set was α = .75.

*Dietary habits* questio*ns* asked (measured by the LILAC Annual Survey), "How many cups of fruit and vegetables do you eat in an average day?" with answers ’Less than 4.5 cups (1)’, or ’4.5 cups or more (2)’, and questions with ’Yes (1)’ or ’No (0)’ answer, asked whether a participant ate fish, and whole grains regularly, and avoid high sugary drink and sodium intake. Then, scores for total questions were summed, and the total dietary habit score was computed (0 ‘poor diet habit’ to 6 ‘ideal diet habit’) [18, 19]. The intraclass correlation of our data set was 0.77 indicating a good test-retest reliability.

*Physical activity* was ascertained based on total physical activity measured by metabolic equivalent/week, measured by the Lifestyle Questionnaire. Women were asked: 1) How often they walked outside the home for more than 10 minutes without stopping (never, 1–3 times each month, 1 time each week, 2–3 times each week, 4–6 times each week, and 7 or more times each week); 2) Duration of each time they walked longer than 10 minutes. Other physical activities were classified as either mild or moderate/severe; and 3) Frequency of activities (days per week: none, 1 day per week, 2 days per week, 3 days per week, 4 days per week, 5 or more days per week) and duration (minutes per week: none, 0–30 minutes, 31–60 minutes, 61–90 minutes, 91–120 minutes, 121–150 minutes, greater than 150 minutes). Metabolic equivalents (METs) were assigned for each type of activity, which was multiplied by the total activity time per week (hr./week) to determine MET-hr./week. Physical activity was analyzed using MET-hr./week for this analysis [22].

### Life impact measures

*HRQOL*, *daily life interferences*, *and body image index* were analyzed as impact variables of GI symptoms. HRQOL and daily life interferences were measured from the Lifestyle Questionnaire. The score of the HRQOL question *(how would you rate your QOL in the past year*) ranged from 0 *(worst)* to 10 *(best))*. Daily life interferences were defined as three sub-concepts: physical function (10 items), independent daily routine function (7 items), and social function (6 items). The daily life interferences were operationalized using the physical function composite score, independent daily routine function composite score, and social function composite score in the past year from the Lifestyle Questionnaire. Scores ranged from 0 to 100, with lower scores indicating less daily life interference. Cronbach’s alpha using our data set was α = .95 for daily life interference.

The body image index was measured with questions from the LILAC Annual Survey. The first question *(‘How satisfied are you with your appearance*?*’)* is scored using a 4-point Likert scale from 0 *(less satisfied with body appearance)* to 3 *(very much satisfied with body appearance)*. The second *(‘Have you felt less physically attractive as a result of your cancer or treatment*?*’)* and third questions *(‘Have you been dissatisfied with the appearance of any scar(s) that resulted from your cancer treatments*?*’)* are reversely coded, resulting in a range of 0 *(more favorable body image)* to 3 *(less favorable body image)*. An averaged body image score (calculated using the first question and reverse scores of the second and third questions) was used. Scores range from 0 to 3, with higher scores indicating a more favorable body image. Cronbach’s alpha using our data set was α = .91.

### Statistical analyses

Descriptive statistics (i.e., mean and standard deviation [SD]; N and %) were used to describe the baseline, cancer characteristic variables, symptom variables, and life impact variables. To identify potential factors relating to overall GI symptoms, we dichotomized the CRC survivors based on a composite GI symptom score (an asymptomatic group ‘if a composite GI score = 0’ versus a symptomatic group ‘if a composite GI score >0’). Then, we used a Chi-square test or a Fisher’s exact test (if > 20% of expected cell counts were < 5) for categorical variables and an independent t-test for continuous variables to compare differences in variables by asymptomatic versus symptomatic GI symptom groups. We used *Pearson’s* correlation coefficient for continuous variables and analysis of variance (ANOVA) for categorical variables of demographic and clinical characteristics to investigate further bivariate correlations between potential correlates and individual GI symptoms. Then, we also used a multivariate linear regression model to explore potential risk and impact factors of GI symptoms. We included hypothesized predictor variables among demographic and clinical characteristics, and non-GI symptoms if it was statistically significantly associated with GI symptoms in the previous analyses aforementioned above. Also, we controlled for race, education, marital status, insurance type, BMI, cancer stage, treatment, time since diagnosis, and comorbidities, that have shown an association with cancer symptoms in previous literature [23].

We investigated distributions of overall data, and a normal distribution with no skew was observed for these variables. Stepwise eliminations were performed using p-values of ≥ 0.05 as the limiting threshold. Correlation analyses between the independent variables and variance inflation factors (VIF) were calculated to assess multicollinearity. Multi-collinearity was present if the mean VIF was greater than 5 for the current study [24]. Stepwise regression was used in our study to resolve the potential multicollinearity among multiple variables. For all analyses in this study, standardized beta coefficients (β) were used to quantify the strength of the associations. All tests were two-sided and statistical significance was defined as a p-value <0.05. All analyses were performed using SPSS 24 (IBM Corp., Armonk, NY, USA).

## Results

### Demographic and clinical characteristics

In the WHI LILAC study, a total of 796 CRC survivors’ data collected between 2011–2015 was available for this study. After excluding those with missing data from the necessary questionnaires of this study, our sample included 413 female CRC survivors (n = 341 in the colon, n = 33 in the rectum, and n = 28 in colorectal cancers). The mean age at diagnosis and the completion of survey forms was 62.7(SD 6.3) and 71.2 (SD 6.3), respectively (Table 1). The majority were non-Hispanic White (89.3), followed by Black (5.1%). Half were married/partnered (42.5%) and attended post-graduate or professional schools (47%). Most participants were diagnosed with CRC (76%) for longer than 5 years and with stage II (59.8%). Time since diagnosis was 8.1 (for mean) and 8.0 (for median) years (range 4.2–15.5). 63.2% of patients underwent surgery and 34.4% of CRC survivors received multiple treatment regimens, including surgery and chemoradiation.

**Table 1:** Demographic and clinical characteristics of LILAC CRC survivors.

Variables		CRC Survivors (N = 413)			
		Total Survivors (N = 413)	Positive GI Symptoms^a^ (N = 333, 81%)	No GI Symptoms^a^ (N = 80, 19%)	*t or F*, *p*^*b*^^,^^*^
N (%) unless otherwise specified					
**Age at diagnosis, yrs. (mean, SD) [range]**		62.7 (6.3), [50, 77]	63.2 (7.2), [50, 77]	62.6 (6.0), [50, 76]	0.622, .762
**Age at completion of survey forms, yrs. (mean, SD) [range]**		71.2 (6.3), [53, 91]	70.6 (7.1), [53, 90]	70.7 (6.2), [53, 91]	0.598, .572
**Weight at screening, lbs (mean, SD)**		153.32(30.1)	155.08(32.0)	152.53(31.0)	-0.637,.886
BMI at screening, kg/m^2^ (mean, SD)		28.4 (5.6)	28.3 (5.4)	28.1 (5.0)	-0.203,.434
**Race**	White	369 (89.3)	299 (89.8)	70 (87.5)	6.765, .149
	Black	21 (5.1)	16 (4.8)	5 (6.3)	
	Asian	12 (2.9)	7 (2.1)	5 (6.3)	
	More than one race or unknown	11 (2.7)	11 (3.3)	0 (0.0)	
**Marital status**	Never Married	16 (4.1)	12 (3.8)	4 (5.1)	0.289, .962
	Married/Partnered	166 (42.5)	126 (40.4)	40 (50.0)	
	Divorced/Separated	52 (13.3)	41 (13.1)	11 (13.9)	
	Widowed	157 (40.2)	133 (42.6)	24 (32.1)	
**Time since diagnosis**	Mean (SD)/	8.1(3.8)/	8.1 (3.9)/	8.1(3.8)/	.890, .943
	Median [range], yrs.	8.0 [4.2, 15.5]	8.1 [4.2, 14.4]	8.1 [4.2,15.5]	
	< 5 years	99 (24.0)	83 (24.9)	16 (20.0)	1.321, .**038**
	≥5 and < 10 years	169 (41.0)	136 (40.8)	33 (41.3)	
	≥ 10 years	145 (35.0)	114 (34.2)	29 (38.8)	
**Stage at diagnosis**	II	156 (37.8)	117 (34.9)	39 (48.8)	6.932, .**049**
	III	247 (59.8)	206 (62.0)	41 (51.2)	
	IV	9 (2.2)	9 (2.7)	0 (0.0)	
**Type of cancer treatment**	Surgery only	261 (63.2)	213 (64.0)	48 (60.0)	5.313, .782
	Multiple Treatments	142 (34.4)	111 (33.3)	31 (38.8)	
	*None underwent radiation only or hormone therapy only. *One patient in the GI-positive symptom group underwent chemotherapy only*.				
**Comorbidities modified Charlson index**	Charlson Index Score	0.9 (1.0)	0.9 (1.0)	0.7 (0.9)	1.861, .411
	Categorized Mild	304 (73.6)	238 (71.4)	66 (82.5)	4.419, .246
	Categorized Moderate	75 (8.2)	66 (19.8)	9 (11.3)	
	Categorized Severe	34 (18.2)	29 (8.7)	5 (6.3)	
**Education**	≤ High school graduate	75 (18.2)	61 (18.4)	14 (17.4)	1.773, .777
	College Graduate	143 (34.8)	115 (34.7)	27 (34.2)	
	Postgraduate/Professional	193 (47.0)	155 (46.8)	38 (48.1)	
**Insurance**	Medicare	160 (38.8)	129 (38.9)	31 (33.4)	1.827, .935
	Medicaid	8 (1.9)	7 (2.1)	1 (1.3)	
	Private or commercial	118 (28.6)	94 (28.3)	24 (30.0)	
	Veterans/State/free care	30 (7.0)	26 (7.8)	4 (5.1)	
	No insurance	97 (23.5)	76 (22.9)	21 (26.3)	
**Household Income/yr.**	< $15,000	34 (8.7)	30 (9.6)	4 (5.2)	5.611, .346
	$15,000 - $50,000	193 (49.2)	148 (47.1)	45 (58.5)	
	> $50,000 - $75,000	87 (22.2)	74 (23.6)	13 (16.9)	
	> $75,000	78 (19.9)	62 (19.7)	16 (19.6)	

^a^ Colorectal cancer (CRC) survivors were dichotomized as an ‘asymptomatic’ GI symptom

group (if a composite GI symptom score = 0) or a ‘symptomatic’ GI symptom group (if a composite GI symptom score >0) in the past 4 weeks.

^b^ independent *t-test* for continuous variables, Chi-square test, or Fisher’s exact test (if > 20% of expected cell counts were < 5) for categorical variables.

* p < .05. Statistically significant values were formatted in bold.

### GI symptoms

**Fig 1:**
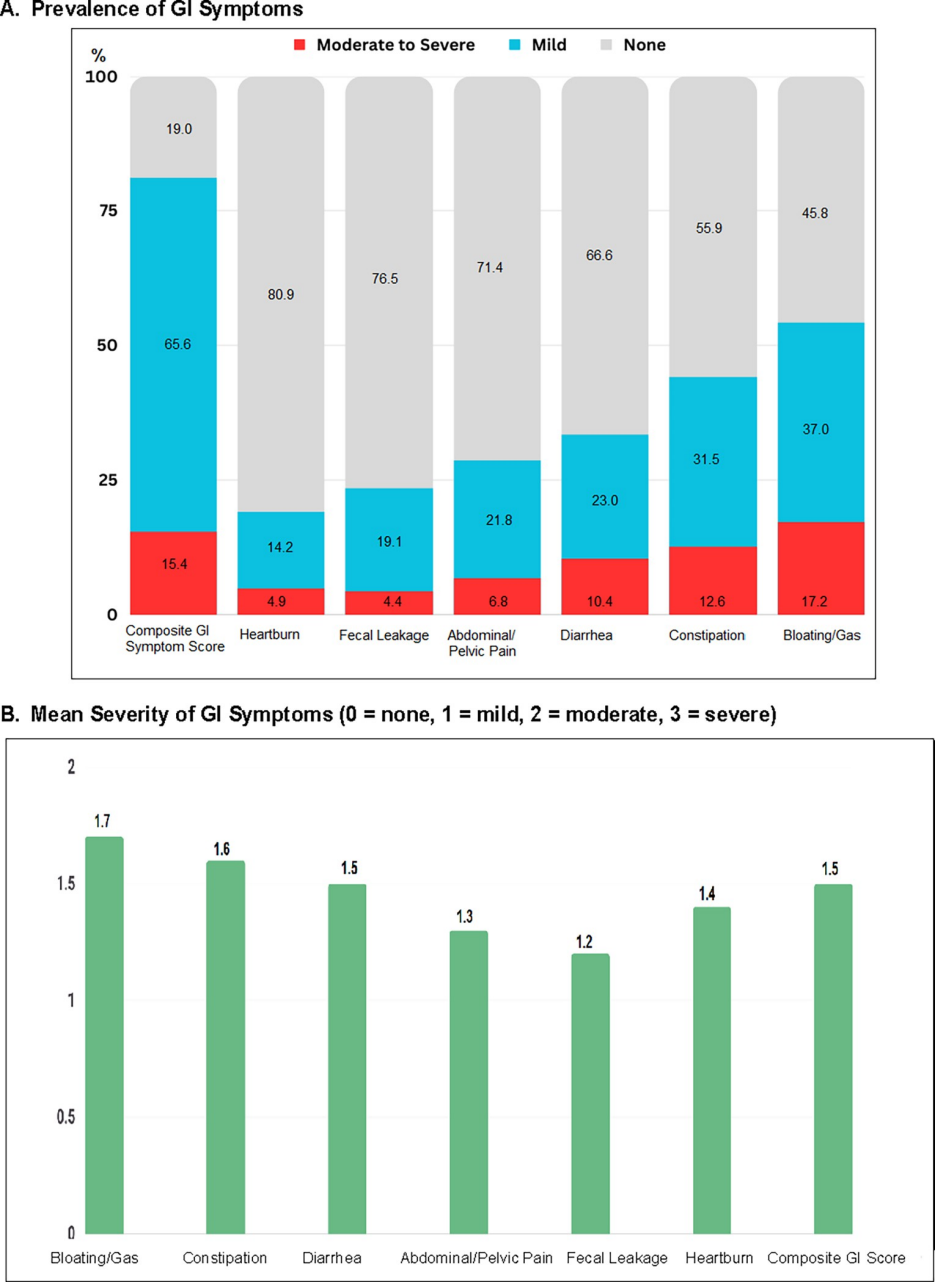
GI symptoms in CRC survivors (Prevalence, and severity). A. Prevalence of GI symptoms with mild (blue bar) and with moderate-to-severe symptoms (red bar). Y-axis unit % (prevalence of GI symptoms among 413 colorectal cancer survivors). B. Mean Severity of GI symptoms (Y axis unit 0 ‘none’ to 3 ‘severe’ Likert point scale). N = 413.

Of 413 CRC survivors in this study, 81% (SD 2.42) of CRC survivors reported GI symptoms (N = 333) with a mean of 8.1 years since cancer diagnosis; and 61% of the total 413 survivors (n = 250) still experienced GI symptoms beyond 5 years after cancer diagnosis (Table 1). Abdominal bloating/gas was the most common (54.2%, SD 0.88) and severe GI symptom (mean = 1.7), followed by constipation (prevalence = 44.1%, SD 1.06; severity mean = 1.6) and diarrhea (prevalence = 33.4%, SD 0.76; severity mean = 1.5) (Fig 1). Of note, 17.2% of patients reported moderate-to-severe abdominal bloating/gas, followed by constipation (12.6% with moderate-to-severe) and diarrhea (10.4% with moderate-to-severe). In a composite GI symptom score, 15.4% of CRC survivors reported moderate-to-severe overall GI symptoms (Fig 1).

### Factors relating to GI symptoms (Bivariate correlations, unadjusted results)

#### Demographic and clinical correlates of GI symptoms

We first compared demographic and clinical characteristics between asymptomatic and symptomatic GI symptom groups through an independent t-test or Chi-square/Fisher’s exact tests (Table 1). Time since diagnosis and cancer stages were significantly different between the asymptomatic and symptomatic GI symptom groups. CRC survivors less than 5 years after cancer diagnosis were more prevalent (24.9%) in the positive GI symptom group than the asymptomatic GI symptom group (20.0%, *p* = 0.038). CRC survivors with stage III were prevalent (62%) in the symptomatic GI symptom group compared to the asymptomatic group (51.2%) (*p* = 0.049) (Table 1).

In the analysis for examining associations of demographic and clinical characteristics with individual GI symptoms (i.e., compare individual GI symptom severity by time since diagnosis categories, and by cancer stage categories) (S1 Table), time since diagnosis was positively associated with some of the individual GI symptoms: bloating/gas (mean severity 1.3 in <5 years, versus 0.7 in ≥ 5 years, *p* = 0.013), constipation (mean 0.9 in <5 years, versus 0.5 in ≥ 5 years, *p* = 0.017), and abdominal/pelvic pain (mean 0.4 in <5 years, versus 0.2 in ≥ 5 years, *p* = .011). Among 6 individual GI symptoms, abdominal pain severity was positively associated with advanced cancer stage (mean pain severity 0.9, 1.0, and 1.4 in stages II, III, and IV, respectively, overall *p* = 0.033). Except for the time since the cancer diagnosis, and cancer stage, none of the demographic or clinical characteristics were significantly associated with individual GI symptoms (S1 Table).

#### Association of GI symptoms with psychological distress, lifestyle factors, and life impact measures

Psychological distress, dietary habits, HRQOL, daily physical function interferences, and body image index significantly differed between the symptomatic and asymptomatic GI symptom groups (Table 2). The severity of depression, anxiety, fatigue, and sleep disturbance was higher in the GI symptom group than in the asymptomatic group. The asymptomatic GI symptom group had healthier dietary habits, better HRQOL, more satisfaction in their body image, and fewer daily interferences in physical function, compared to the symptomatic group. In *Pearson* correlation analyses (Table 3), psychological distress, lifestyle, and life impact variables were also significantly associated with GI symptoms. Of note, fatigue was the most strongly related to a composite GI symptom score (*r* = 0.298, *p* = .004). Sleep disturbance showed a significant relationship with bloating/gas (*r =* 0.200, *p* = .002), diarrhea (*r* = 0.192, *p* = .005), fecal leakage (*r* = 0.135, *p* = .001), and abdominal/pelvic pain (*r* = 0.236, *p* = .003). Healthier dietary habits and increased physical activity levels were associated with lower constipation (*r =* -0.103, *p* = .04; *r =* -0.041, *p* = .01, respectively) and lower diarrhea (*r* = -0.123, *p* = .04; *r* = -0.121, *p* = .03, respectively). In daily life interferences, none of the GI symptoms interferes with independent daily routine function, while several GI symptoms interfere with daily physical and social functions. Specifically, abdominal pain severity was positively correlated with severe daily interferences in physical function (*r* = 0.274, *p* = .001) and social function (*r* = 0.134, *p* = .003); and negatively correlated with body image satisfaction (*r* = -0.180, *p* = .004).

**Table 2:** Psychological distress, lifestyle, and life impact variables in CRC survivors.

Variables (Mean, Standard Deviation)	Total Survivors (N = 413)	Positive GI Symptoms^a^ (N = 333)	No GI Symptoms^a^ (N = 80)		*t*, *p*^*b*^^,^^*^
**Psychological distress (0 ‘none’ to 3 ‘severe’) in the past 4 weeks**					
Depression	0.4 (0.5)	0.5 (0.6)		0.2 (0.4)	-2.910, **< .001**
Anxiety	0.5 (0.7)	0.6 (0.7)		0.2 (0.5)	-3.477, **< .001**
Fatigue	1.0 (0.7)	1.2 (0.7)		0.7 (0.7)	-4.337, **< .001**
Sleep disturbance	0.8 (0.8)	0.9 (0.9)		0.4 (0.6)	-4.68, .**005**
**Dietary Habits (0 ‘poor diet habit’ to 6 ‘ideal diet habit’)**					
	2.9 (1.1)	2.6 (1.1)		3.5 (1.0)	1.702, .**045**
**Physical Activity Level (Total MET-hr./week)**					
	8.9 (9.0)	8.5 (8.7)		10.4 (9.6)	1.574, .220
**Life Impact Variables**					
Health-Related Quality of life (Rating 0 ‘worst’ to 10 ‘best’)	7.9 (1.3)	7.8 (1.0)		8.4 (1.4)	3.888, .**007**
Daily life interferences (rating 0 ‘no interference’ to 100 ‘severe interference’)					
Physical function	69.7 (24.1)	75.3 (23.1)		68.4 (24.1)	1.108, **.028**
Independent daily routine function	40.0 (0.6)	40.1 (0.9)		40.0 (0.5)	2.231, .571
Social function	38.1 (7.4)	38.8 (7.0)		37.9 (7.5)	0.962, .208
Body Image Index (Rating 0 ‘less satisfaction’ to 100 ‘more satisfaction’)	67.5 (0.3)	66.4 (0.3)		69.6 (0.2)	3.186, **.002**

^a^ Colorectal cancer (CRC) survivors were dichotomized as an ‘asymptomatic’ GI symptom

group (if a composite GI symptom score = 0) or a ‘symptomatic’ GI symptom group (if a

composite GI symptom score >0) in the past 4 weeks.

^b^ independent *t-test* for continuous variables.

* p < .05. Statistically significant values were formatted in bold.

**Table 3:** Bivariate correlations of GI symptoms with psychological distress, lifestyles, and life impact variables.

Variables	Composite GI Symptom score	Bloating/Gas	Constipation	Diarrhea	Fecal leakage		Abdominal/Pelvic pain	Heartburn
**Psychological Distress (0 ‘none’ to 3 ‘severe’) in the past 4 weeks**								
Depression	0.166**	0.103*	0.146**	0.097*	0.114**	0.081		0.030
Anxiety	0.228**	0.203**	0.140**	0.111**	0.040	0.140**		0.148**
Fatigue	0.298**	0.253**	0.137**	0.138**	0.105*	0.234**		0.181**
Sleep disturbance	0.281**	0.200**	0.093	0.192**	0.135**	0.236**		0.139**
**Dietary Habits (0 ‘poor diet habit’ to 6 ‘ideal diet habit’)**								
	-0.122*	-0.054	-0.103*	-0.123*	0.023	-0.040		-0.063
**Physical Activity Level (Total MET-hr./week)**								
Total energy expenditure/wk., MET-hrs.	-0.022	-0.123*	-0.041*	-0.121*	-0.007	-0.032		-0.098*
**Life Impact Variables**								
HRQOL (0‘worst’ to 10 ‘best’)	-0.232**	-0.144**	-0.163**	-0.163**	-0.131**	-0.148**		-0.059
Daily Life Interferences (rating 0 ‘no interference’ to 100 ‘severe interference’)								
Physical function	0.202**	0.109**	0.179**	-0.065	0.148**	0.274**		-0.044
Daily routine function	-0.022	-0.033	-0.048	0.017	-0.053	0.007		0.041
Social function	0.140**	-0.045	-0.095	-0.050	-0.092	0.134**		0.099*
Body Image Index (0 ‘less satisfaction’ to 100 ‘more satisfaction’)	-0.201**	-0.155**	-0.042	-0.131**	-0.148**	-0.180**		-0.062

* p < .05, ** p < .01 based on *Pearson* correlation analysis. The model was unadjusted for covariates. Health-Related Quality of Life **=** HRQOL.

#### Potential risk factors and life impact of GI symptoms

Using multivariate regression models adjusted for covariates aforementioned above (race, education, marital status, insurance type, BMI, cancer stage, treatment, time since diagnosis, and co-morbidities), we explored potential risk factors and the impact of GI symptoms. Both fatigue and sleep disturbance were important risk factors related to several GI symptoms (bloating/gas, abdominal/pelvic pain), and a composite GI symptom score. Sleep disturbance was associated with diarrhea and fecal leakage (Table 4). For every additional degree of symptom severity, the expected severity of a composite GI symptom score significantly increased by 0.65 (B = 0.65, *p* < .001) for fatigue, and 0.54 (B = 0.54, *p =* 0.001) for sleep disturbance on average, assuming other variables were constant. Among potential risk factors including psychological distress and lifestyle factors (diet and physical activity), fatigue was the greatest risk factor for GI symptoms (β = 0.21, *p* < .001) for a composite GI symptom score and a bloating/gas symptom. Furthermore, depression was a significant risk factor for constipation (β = 0.17, *p* = 0.016), and healthier dietary habits were associated with lower severity of diarrhea (β = -0.12, *p* = 0.008). In terms of the life impact of GI symptoms, altered bowel patterns (constipation or diarrhea) and abdominal/pelvic pain negatively impacted HRQOL. Of note, abdominal/pelvic pain was the greatest impact factor of HRQOL (β = -0.13, *p* = .003), interferences in physical and social functions (βs = 0.13, *p* = .008, and *p* = .007, respectively), and body image satisfaction (β = -0.18, *p* = .003).

**Table 4:** Correlates of GI symptoms (Potential risk and impact factors).

	Unstandardized B (95% CI)	*SE*	Standardized β	t, p*
**Potential Risk Factors for GI Symptoms (0 no to 3 severe)**				
Composite GI Symptom Score				
Fatigue	0.65 (0.29, 0.99)	.18	0.21	3.557, < .001
Sleep Disturbances	0.54 (0.22, 0.84)	.16	0.20	3.336,. < .001
Bloating/Gas				
Fatigue	0.22 (0.09, 0.34)	.06	0.21	03.468, .001
Sleep Disturbances	0.13 (0.02, 0.23)	.05	0.14	2.242, .006
Constipation				
Depression	0.17 (0.03, 0.31)	.07	0.15	02.415, .016
Diarrhea				
Sleep Disturbances	0.16 (0.06, 0.25)	.05	0.19	3.245, .001
Dietary Habits	-0.08 (-0.15, -0.01)	.03	-0.12	-2.088, .008
Fecal Leakage				
Sleep Disturbances	0.09 (0.01, 0.17)	.04	0.14	2.233, .026
Abdominal/Pelvic Pain				
Fatigue	0.13 (0.04, 0.22)	.04	0.18	2.947, .003
Sleep Disturbances	0.12 (0.04, 0.19)	.04	0.18	2.994, .003
Heartburn				
Fatigue	0.14 (0.05, 0.24)	.04	0.18	3.026, .003
Life Impact of GI Symptoms				
Health Related-Quality of Life (rating 0 ‘worst’ to 10 ‘best’)				
Constipation	-0.24 (-0.46, -0.02)	.11	-0.13	-2.171, .001
Diarrhea	-0.24 (-0.45, -0.02)	.11	-0.12	-2.098 .027
Abdominal/pelvic pain	-0.24 (-0.45, -0.02)	.11	-0.13	-2.012, .003
Daily Life Interferences (rating 0 ‘no interference’ to 100 ‘severe interference’)				
Interferences in Physical Function				
Abdominal/pelvic pain	5.61 (4.31, 15.9)	.23	0.13	2.386, .008
Constipation	3.72 (3.45, 10.5)	.18	0.11	2.052, .021
Interferences in Social Function				
Abdominal/pelvic pain	1.82 (1.6, 5.1)	.81	0.13	2.222, .007
Body Image Index (rating 0 ‘less satisfaction’ to 100 ‘more satisfaction’)				
Abdominal/pelvic pain	-0.11 (-0.18, -0.04)	.34	-0.18	-3.002, .003

A linear regression model was adjusted for race, education, marital status, insurance type, BMI, cancer stages, treatment types, time since diagnosis, and co-morbidities.

None of the GI symptoms impacted the interferences of daily routine function.

* p < .05 indicates statistically significant values.

## Discussion

CRC survivors often experience prevalent and severe long-term GI symptoms after cancer treatments. This is the first study to address a significant knowledge gap in the linkage of GI symptoms with potential risk factors and adverse life impacts. Our findings provide information about who is at risk and factors contributing to high risk for GI symptoms such as lifestyle factors, and psychological distress. Given the significant impact of GI symptoms on multiple life impact measures in our study, our findings suggest the need to consider GI symptom management in long-term CRC cancer survivorship. This study will help identify individualized interventions in preventing and managing GI symptoms.

Our study leveraged the collection of self-reported GI symptoms in CRC survivors within the existing WHI LILAC study addressing symptoms among patients with multiple cancer types [23]. In a previous WHI LILAC cancer symptom study [23], only 10% of CRC survivors were included and no GI symptom was included except for dry mouth symptom. Furthermore, our study expands the previous findings of GI symptoms in CRC survivors within one or two years after cancer diagnosis in men majority of patients [3, 14]. The results of the current study demonstrated that the majority of CRC survivors (81%) have a substantial long-term GI symptom burden. The GI symptoms in CRC survivors (specifically bloating/gas, constipation, diarrhea, and abdominal/pelvic pain) may persist beyond 10 years after a cancer diagnosis. Our study also showed that psychological distress (specifically fatigue and sleep disturbance) was the most important factor in long-term GI symptoms. GI symptoms impacted HRQOL, physical and social functions, and body image. Abdominal pain was the major GI symptom, which caused distress and deteriorated function and HRQOL of long-term CRC survivorship.

Overall, CRC survivors in our study showed relatively higher prevalence and severity of GI symptoms compared to previous studies in other cancers [25, 26] and CRC [3, 14]. In Rietveld et al. study of 131 women with ovarian cancer [25], only 25% of OC survivors reported GI symptoms (81% in our study). In a review of breast cancer symptoms, GI symptoms were not included within the top 14 frequent and severe symptoms in breast cancer survivors including cognitive impairment, fatigue, sexual dysfunction, neuropathy, pain, lymphedema, mouth sore, urinary incontinence, etc. [26]. In 447 Irish CRC survivors (37% female) less than 1–3 years after the cancer diagnosis, abdominal bloating/gas (25.5% with mild to severe) was the most frequent GI symptoms followed by fecal incontinence (23.5%), and female sex, radiation and cancer stage were associated with several GI symptoms [3]. In 474 CRC survivors (49% female) approximately 4 years after cancer diagnosis in the US, diarrhea (13%) and constipation (7%) were the most commonly reported GI symptoms [14]. These differences could be due to a sex effect (women experienced and reported more symptoms than men [27]), different sample sizes, heterogeneity of cancer-related factors such as cancer stage, or types of cancer treatments across the studies.

Age, comorbidity, income, social support, race, and cancer stage were associated with symptom burden in many cancer types [4, 28]. However, time since diagnosis and cancer stages were only significantly related to GI symptoms in our study. Specifically for the time since diagnosis, our result indicates that CRC survivors with less than 5 years after cancer diagnosis might have more severe GI symptoms than CRC survivors with 5 or more years after diagnosis. The advanced cancer stage was positively associated with a composite GI symptom score and abdominal pain. This is consistent with previous research showing associations between the advanced cancer stage and overall bodily pain in most cancer types [29]. Since cancer-related distress was shown to be higher in CRC survivors with advanced cancer stages [12], the high-stress levels in CRC survivors with advanced cancer stage might trigger stress-induced visceral pain by activating glucocorticoid receptor and corticotropin-releasing hormone-mediated mechanisms in the amygdala involved in stress-induced visceral hypersensitivity [30].

We found that CRC survivors who experienced GI symptoms reported significantly more psychological distress including fatigue, sleep disturbances, depression, and anxiety, than those without GI symptoms. In previous studies of CRC survivors, fatigue and other psychological distress were the most common symptoms [3–5, 14]. Building upon prior work, our findings contribute to the literature by demonstrating strong relationships between GI symptoms and psychological symptoms. It is possible to consider bidirectional relationships between GI symptoms and psychological distress (e.g., GI symptoms disturbed sleep quality and increased fatigue). Increasing evidence suggests the emerging role of the microbiome in chemo or radiation treatments-induced late symptom toxicities (psychological distress, GI symptoms) under the brain-gut axis [31, 32]. The link between GI and psychological symptoms was demonstrated in many chronic diseases including irritable bowel syndrome, chronic fatigue syndrome, and inflammatory bowel disease [32]. In a review of the brain-gut axis in GI cancers [33], a significant association was found between gut microbiota dysfunction and psychological distress. Gut dysbiosis by cancer treatment or cancer per se in the GI tract can lead to systemic inflammation. The increased pro-inflammatory system carried by gut dysbiosis can reach the brain directly via the bloodstream or other indirect transmission pathways, which leads to psychological distress [33]. In contrast, chronic psychological distress can lead to gut dysbiosis and long-term GI symptoms by exacerbated gut permeability, sensitivity, and motility through the brain-gut axis with mucosal immune activation, peripheral neurons, and gut microbiota, and systemic inflammations [4, 34, 35]. Our findings implicate that GI and psychological distress can co-occur in the long term in CRC survivors, or CRC survivors with GI symptoms are more prone to feel psychological distress or vice versa. However, given the cross-sectional study design of this study, the causal effect between GI and psychological symptoms is unknown.

Patients with more depression also reported frequent and severe constipation. Potential factors relating to depression such as lack of physical activity, dehydration, poor appetite, or use of anti-depressants agents may contribute to constipation. While lifestyle was not a significant risk factor of most GI symptoms in our study, our findings still showed the need for lifestyle management (food, and exercise) specifically for constipation and diarrhea management.

Abdominal pain/pelvic pain was the greatest GI symptom, affecting daily life interferences in physical and social functions, and body image satisfaction. Pain is one of the main disturbing symptoms with a major impact on functional capacity and HRQOL in other cancer types [15]. Previous research on CRC survivors [5] showed that the frequency and severity of abdominal pain was higher and more severe than general pain. This result proposes a potential role of gut biological factors such as gut microbiome or metabolites and inflammatory exacerbation in CRC survivors, which could differ from other cancer types. Further investigation of underlying mechanisms including biological factors is warranted.

### Implication for practice

A recent evidence-based guideline on managing GI symptoms indicated that the comprehensive assessment and management of GI symptoms are overlooked, and a validated GI symptom assessment tool is not yet available [36, 37]. Currently, GI symptom management primarily depends on pharmacologic treatments in CRC survivors. No existing evidence-based non-pharmacological interventions focus on GI symptoms in this population. Our findings shed light on the importance of psychosocial support as well as lifestyle interventions (specifically nutritional management) in managing GI symptoms in CRC survivors. In our study, lifestyle was associated with GI symptoms, but this association became weaker when we adjusted covariates, and psychological factors remained as the most significant risk factors for GI symptoms. Psychological distress would mediate the association between lifestyle and GI symptoms. Thus, here, we emphasize the critical need for early psychosocial interventions to alleviate GI symptoms in this patient group. We also suggest individualized nutritional counseling for improving diet quality, particularly, among patients at high risk of psychological distress, or altered bowel patterns. Furthermore, our results highlight the need for physical rehabilitation programs to be integrated into survivorship care to maintain daily function during cancer treatment and the long-term follow-up phase.

### Strengths and limitations

The strengths of our study leveraged longitudinal data (with up to 20 years of follow-up after cancer diagnosis), so that we could characterize persistent GI symptoms after cancer diagnosis/treatment, specific to female CRC survivors. Limitations of our study include a cross-sectional study design, which prevented us from identifying causal effects between risk factors, life impact, and GI symptoms, and changes in GI symptoms over time are unknown. Furthermore, our study samples were limited to women registered in the WHI OS and ES2. Thus, the results may not be generalized to other populations, including men CRC survivors. Even though self-reported symptom data can provide insight into individuals’ subjective experiences, individuals may underreport or overreport symptoms. They may not accurately remember or report their symptoms in our study. The validated cancer symptom assessment instruments are warranted to mitigate the biases of self-reported symptom data., e.g., MD Anderson Symptom Inventory (MDASI), Memorial Symptom Assessment Scale (MSAS), and European Organization for Research and Treatment of Cancer Quality of Life Questionnaire Colorectal Module (EORTC-QLQ CR38 and CR29) [38]. Lastly, controlling for covariates may not completely eliminate the impact of confounding variables, such as unmeasured comorbidities.

## Conclusions

We report significantly persistent high GI symptoms in female CRC survivors, even in remission. We propose to specifically consider individualized psychosocial support, lifestyle management, pain management, and physical rehabilitation for long-term CRC survivors. It is also essential to have policies and programs that can support these interventions to improve CRC women’s physical and emotional health (e.g., policymakers can implement these programs to increase access to such interventions and support survivorship care). Healthcare professionals can integrate these interventions into clinical practice to improve the survivors’ QOL as well. A longitudinal study may be needed to better understand the changes in GI symptoms over time and to determine the causal effect of GI symptoms. Further research is needed to examine the underlying biological mechanisms of GI symptoms.

## Supporting information

S1 Table Bivariate correlations (unadjusted results) of demographic and clinical data with individual GI symptom severity (N = 413). (DOCX)
