# Supplementary material for: Factors associated with long-term gastrointestinal symptoms in colorectal cancer survivors in the women’s health initiatives (WHI study)
Source: PLoS One. 2023 May 19;18(5):e0286058. doi: 10.1371/journal.pone.0286058 (PMC10198480; doi:10.1371/journal.pone.0286058)
Supplement: S1 Table — (DOCX) [file pone.0286058.s001.docx]

**Supplementary Table 1. Bivariate Correlations (unadjusted results) of Demographic and Clinical Data with Individual GI Symptom Severity (N = 413).**

|  | | Composite  GI Symptom score | | Bloating/Gas | | Constipation | | Diarrhea | | Fecal leakage | | Abdominal/  Pelvic pain | | Heartburn | |  |
| --- | --- | --- | --- | --- | --- | --- | --- | --- | --- | --- | --- | --- | --- | --- | --- | --- |
| Data are presented as the Pearson Correlations *r* and *p* | | | | | | | | | | | | | | | |  |
| Age at diagnosis, yrs. | | .007 | .895 | -.021 | .675 | .015 | .762 | .008 | .879 | .036 | .463 | -.023 | .643 | -.058 | .238 |  |
| Age at completion of survey forms, yrs. | | -.024 | .622 | -.046 | .354 | .999 | .999 | -.005 | .921 | .096 | .051 | -.050 | .311 | -.075 | .130 |  |
| Weight at screening, lbs | | .092 | .061 | .085 | .412 | .072 | .145 | .008 | .879 | .036 | .463 | .041 | .407 | .088 | .119 |  |
| BMI at screening, kg/m^2^ | | -.059 | .229 | .040 | .412 | .047 | .132 | .014 | .777 | .052 | .287 | .047 | .341 | .070 | .156 |  |
| Mean years since cancer diagnosis | | -.059 | .229 | -.033 | .498 | -.027 | .581 | -.058 | .239 | -.023 | .644 | -.056 | .257 | -.011 | .823 |  |
| Data are presented as the mean (SD), F and *p* based on the One-way Analysis of Variance (ANOVA) | | | | | | | | | | | | | | | |  |
| Race | White | 3.0 (0.5) | 1.356, .211 | 1.1 (0.8) | 1.635, .243 | 0.6 (0.8) | 1.222, .121 | 0.5 (0.5) | 1.222, .342 | 0.5 (0.1) | 1.444, .188 | 0.6(0.1) | 1.322,  .183 | 0.5 (0.4) | .212,  .835 |  |
|  | Black | 2.9 (0.4) |  | 1.0 (0.9) |  | 0.6 (0.9) |  | 0.6 (0.8) |  | 0.4 (0.4) |  | 0.4(0.4) |  | 0.5 (0.6) |  |  |
|  | Asian | 2.7 (0.3) |  | 1.2 (1.1) |  | 0.7 (0.6) |  | 0.7 (0.9) |  | 0.4 (0.8) |  | 0.3(0.4) |  | 0.6 (0.5) |  |  |
|  | More than one race or unknown | 2.8 (0.2) |  | 1.2 (1.2) |  | 0.8 (0.6) |  | 0.5 (0.9) |  | 0.5 (0.5) |  | 0.3(0.2) |  | 0.6 (0.5) |  |  |
| Marital status | Never Married | 3.0 (0.6) | 1.437, .231 | 1.1 (0.9) | 1.604, .188 | 0.6 (0.7) | 0.007, .999 | 0.6 (0.6) | 1.135, .335 | 0.6(0.2) | 1.561, .198 | 0.5(0.2) | 1.571, .196 | 0.4(0.6) | .036,  .991 |  |
|  | Married/Partnered | 2.6 (0.3) |  | 0.6 (0.8) |  | 0.6 (0.8) |  | 0.5 (0.7) |  | 0.3(0.6) |  | 0.3(0.5) |  | 0.4 (0.6) |  |  |
|  | Divorced/Separated | 2.8 (0.4) |  | 0.8 (0.8) |  | 0.6 (0.7) |  | 0.6 (0.8) |  | 0.3(0.7) |  | 0.2(0.5) |  | 0.4 (0.6) |  |  |
|  | Widowed | 2.6 (0.1) |  | 0.7 (0.7) |  | 0.6 (0.7) |  | 0.4 (0.6) |  | 0.3(0.4) |  | 0.2(0.3) |  | 0.4 (0.6) |  |  |
| Time since diagnosis |  |  |  |  |  |  |  |  |  |  |  |  |  |  |  |  |
|  | < 5 years | 2.9 (0.6) | **12.**  **384, <.001** | 1.3(0.9) | **4.889, .013** | 0.9 (0.8) | **6.271, .017** | 0.8 (0.9) | **5.789, .017** | 0.4(0.7) | 1.071, .301 | 0.4(0.8) | **6.553, .011** | 0.5 (0.8) | 1.925, .166 |  |
|  | >5 years | 1.6 (0.6) |  | 0.7(0.7) |  | 0.5(0.7) |  | 0.4 (0.7) |  | 0.3(0.5) |  | 0.2(0.5) |  | 0.4 (0.6) |  |  |
| Stage at diagnosis | II | 2.6(0.1) | .665,  .574 | 0.8(0.5) | .085, .968 | 0.6 (0.7) | .808, .490 | 0.4 (0.6) | .554, 646 | 0.5(0.3) | .864, .460 | 0.9(0.5) | **3.412, .003** | 0.5 (0.5) | .173,  .915 |  |
|  | III | 2.7(0.2) |  | 0.7(0.9) |  | 0.5 (0.7) |  | 0.4 (0.5) |  | 0.6(0.5) |  | 1.0(0.4) |  | 0.6 (0.4) |  |  |
|  | IV | 2.9 (0.8) |  | 0.6(0.4) |  | 0.8 (0.8) |  | 0.6 (0.7) |  | 0.5(0.2) |  | 1.4(0.2) |  | 0.5 (0.5) |  |  |
| Type of cancer treatment | Surgery only | 2.1 (0.5) | .423,  .523 | 0.9 (0.4) | .112, .499 | 0.5 (0.6) | .132, .432 | 0.5 (0.6) | .533, 892 | 0.6 (0.2) | .743, .223 | 0.2(0.5) | 1.231, .483 | 0.3 (0.4) | .153,  .875 |  |
|  | Multiple Treatments | 1.9 (0.3) |  | 0.8 (0.5) |  | 0.6 (0.8) |  | 0.5(0.8) |  | 0.5 (0.3~~)~~ |  | 0.2(0.4) |  | 0.3 (0.2) |  |  |
| Comorbidities modified Charlson index | Charlson Index Score | 2.1 (0.4) | .712,  .112 | 1.1 (0.6) | .563, .332 | 0.8 (0.5) | .812, .655 | 0.6 (0.8) | .712, .413 | 0.3 (0.5) | .983, .273 | 0.3(0.4) | .523, .422 | 0.6 (0.5) | 1.321, .523 |  |
|  | Categorized Mild | 1.8 (0.4) |  | 0.9 (0.5) |  | 0.8 (0.4) |  | 0.4 (0.5) |  | 0.4 (0.7) |  | 0.2(0.7) |  | 0.4 (0.5) |  |  |
|  | Categorized Moderate | 2.5 (0.5) |  | 1.3 (0.6) |  | 0.7 (0.6) |  | 0.5 (0.6) |  | 0.6 (0.6) |  | 0.3(0.6) |  | 0.7 (0.6) |  |  |
|  | Categorized Severe | 2.1 (0.6) |  | 1.4 (0.9) |  | 0.9 (0.6) |  | 0.6 (0.7) |  | 0.5 (0.7) |  | 0.3(0.5) |  | 0.6 (0.5) |  |  |
| Education | ≤ High school graduate | 2.9 (2.2) | 1.332, .260 | 1.5(0.5) | 1.051, .847 | 1.3 (0.89) | 1.331, .257 | 0.8 (0.9) | .532, .712 | 0.5 (0.5) | .588, .671 | 0.3(0.5) | .346, .847 | 0.8 (0.8) | 2.043,  .088 | |
|  | College Graduate | 2.9 (2.4) |  | 0.8(1.3) |  | 0.6 (0.7) |  | 0.5 (0.8) |  | 0.4 (0.5) |  | 0.2(0.4) |  | 0.5 (0.7) |  |  |
|  | Postgraduate/Professional | 2.6 (2.4) |  | 0.8(0.7) |  | 0.6 (0.7) |  | 0.5 (0.6) |  | 0.3 (0.5) |  | 0.3(0.5) |  | 0.5 (0.6) |  |  |
| Insurance | Medicare/Medicaid, or Private/commercial or Veterans/State/free care insurance | 2.6 (0.3) | .370., 898 | 0.8(0.5) | 1.091, .367 | 0.7 (0.8) | .222, .969 | 0.8 (0.7) | 1.012, .417 | 0.7(0.5) | .594, .735 | 0.3(0.6) | .700, .650 | 0.6 (0.5) | .726, .629 |  |
|  | No insurance | 2.6 (0.3) |  | 0.6(0.8) |  | 0.6 (0.7) |  | 0.8 (0.5) |  | 0.6(0.6) |  | 0.2(0.5) |  | 0.4 (0.6) |  |  |
| Household Income/yr. | < $15,000 | 3.0 (0.6) | 1.237,.291 | 1.1(0.7) | 1.260, .280 | 0.9 (0.3) | .689, .632 | 0.4 (0.2) | .135, .984 | 0.7(0.2) | .480, .791 | 0.6(0.3) | 1.482, 131 | 0.7 (0.3) | .590, .707 |  |
|  | $15,000 - $50,000 | 2.2 (0.5) |  | 0.9(0.9) |  | 0.6 (0.2) |  | 0.5 (0.2) |  | 0.5(0.1) |  | 0.6(0.7) |  | 0.5 (0.1) |  |  |
|  | > $50,000 -$75,000 | 2.5 (0.4) |  | 0.6(0.7) |  | 0.5 (0.1) |  | 0.5 (0.1) |  | 0.6(0.1) |  | 0.7(0.8) |  | 0.6 (0.1) |  |  |
|  | > $75,000 | 2.3 (0.4) |  | 0.6(0.7) |  | 0.6 (0.7) |  | 0.5 (0.1) |  | 0.5(0.5) |  | 0.8(0.7) |  | 0.6 (0.3) |  |  |

Note. p <.05. Statistically significant values were formatted in bold.
